# A Perspective on the Glass Transition and the Dynamics of Polyelectrolyte Multilayers and Complexes

**DOI:** 10.1021/acs.langmuir.3c00974

**Published:** 2023-10-11

**Authors:** Hongwei Li, Suvesh Manoj Lalwani, Chikaodinaka I. Eneh, Tamunoemi Braide, Piotr Batys, Maria Sammalkorpi, Jodie L. Lutkenhaus

**Affiliations:** † Artie McFerrin Department of Chemical Engineering, Texas A&M University, College Station, Texas 77843, United States; ‡ Jerzy Haber Institute of Catalysis and Surface Chemistry, Polish Academy of Sciences, Niezapominajek 8, 30-239 Krakow, Poland; § Department of Chemistry and Materials Science, Aalto University, P.O. Box 16100, 00076 Aalto, Finland; ∥ Department of Bioproducts and Biosystems, Aalto University, P.O. Box 16100, 00076 Aalto, Finland; ⊥ Academy of Finland Center of Excellence in Life-Inspired Hybrid Materials (LIBER), Aalto University, P.O. Box 16100, 00076 Aalto, Finland; # Department of Materials Science and Engineering, Texas A&M University, College Station, Texas 77840, United States

## Abstract

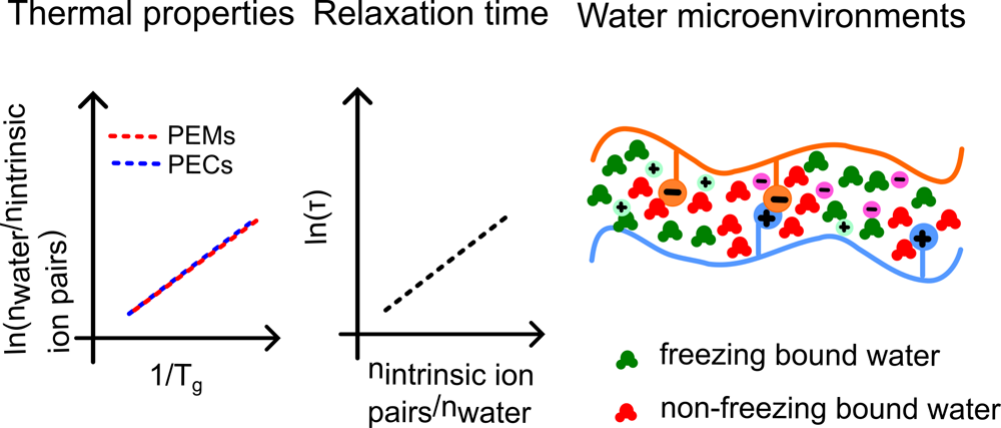

Polyelectrolyte multilayers (PEMs) or polyelectrolyte complexes (PECs), formed by layer-by-layer assembly or the mixing of oppositely charged polyelectrolytes (PEs) in aqueous solution, respectively, have potential applications in health, energy, and the environment. PEMs and PECs are very tunable because their structure and properties are influenced by factors such as pH, ionic strength, salt type, humidity, and temperature. Therefore, it is increasingly important to understand how these factors affect PECs and PEMs on a molecular level. In this Feature Article, we summarize our contributions to the field in the development of approaches to quantify the swelling, thermal properties, and dynamic mechanical properties of PEMs and PECs. First, the role of water as a plasticizer and in the glass-transition temperature (*T*_g_) in both strong poly(diallyldimethylammonium)/poly(sodium 4-styrenesulfonate) (PDADMA/PSS) and weak poly(allylamine hydrochloride)/poly(acrylic acid) (PAH/PAA) systems is presented. Then, factors influencing the dynamics of PECs and PEMs are discussed. We also reflect on the swelling of PEMs in response to different salts and solvent additives. Last, the nature of water’s microenvironment in PEMs/PECs is discussed. A special emphasis is placed on experimental techniques, along with molecular simulations. Taken together, this review presents an outlook and offers recommendations for future research directions, such as studying the additional effects of hydrogen-bonding hydrophobic interactions.

## Introduction

Polyelectrolyte multilayers (PEMs) or polyelectrolyte complexes (PECs) are formed by the spontaneous association of oppositely charged polyelectrolytes (PEs) in aqueous solution, which can be influenced by noncovalent interactions.^1,2^ Wide interest exists due to their remarkable potential as advanced functional materials with applications in drug delivery,^3−5^ sensors,^6−8^ batteries,^9−13^ water treatment,^14−17^ and more. The physical properties of PEMs and PECs can be manipulated by varying the assembly solution pH, ionic strength, salt type, and temperature,^18−30^ which can yield a range of thermal and mechanical properties. However, only recently have we begun to understand these properties due to new advancements in theoretical and experimental approaches. Discussed herein, the past few years have brought discoveries that reveal that nature of the glass transition, the dynamic response, and the important role of water in PECs and PEMs.

In the 1960s, Alan Michaels and co-workers^31−34^ undertook several investigations to determine the interaction characteristics and properties of PECs, for which the kinetics of the association were evaluated in terms of chain conformation and salt type. Later in 2005–2006, Möhwald and Köhler et al.^35,36^ reported that elevated temperature could cause the disruption of adjacent ionic bonds inside PEM capsules, allowing for the rearrangement of oppositely charged polymers. As a result, PEM capsules would rupture or shrink upon heating, depending on the force experienced by the capsule (electrostatic vs hydrophobic). This rearrangement was further supported with evidence of a thermal event that was termed a “melting transition” using differential scanning calorimetry measurements. Later, Imre et al.^37^ assigned the thermal event as the glass transition for hydrated PE assemblies.

The nature of the glass transition for a PEC and PEM is a complex phenomenon that involves not only the disruption of “intrinsic” polycation–polyanion cross-links but also the large-scale macromolecular relaxation of the polymers themselves. The diffusion of polyelectrolytes has been described theoretically using models of “sticky” reptation and the “sticky” Rouse model. The sticky Rouse model was developed by Rubenstein and Semenov^38^ to describe the dynamics of unentangled PECs, whose interaction lifetime is related to the nature of the polycation–polyanion intrinsic ion pair. The sticky reptation model captures the reptation behavior of entangled polyelectrolyte chains, corresponding to conditions at high polymer concentration^39^ and chain length.^40^ A PEC’s glass-transition temperature (*T*_g_)^41^ has been described by the Fox equation, but as discussed herein, more generalized relationships have resulted.

As Michaels stated in 1965,^33,34^ exposure to water and salt can soften PECs. This indicates their important role in changing the PEC’s dynamics. In the early 2000s, water and salt were revisited as a swelling agent and as a plasticizer in both PECs and PEMs. Depending on the polyelectrolyte choice and the number of intrinsic ion pairs (as influenced by salt and pH), the swelling of a PEC or PEM can be tuned.^42^ For example, doping a PEC or PEM with small salt ions breaks intrinsic ion pairs and generates extrinsic ion pairs, thus decreasing the degree of complexation and increasing the PEC or PEM’s ability to swell. Water and salt were leveraged by the Schlenoff group in 2012 to yield “saloplastics”,^43,44^ or hydrated PECs that could be molded and extruded at temperatures above a salt-dependent thermal transition. Elsewhere, water has yielded softening and self-healing behavior in PECs and PEMs.^45−49^ This behavior has been attributed to water plasticization, which lowers the *T*_g_.^43,50^ However, at that time, a quantified perspective of how and why water influences the swelling, the glass-transition temperature, and the dynamics of PECs and PEMs was lacking.

In this Feature Article, we summarize our contributions to the field in developing approaches to quantify the swelling, thermal properties, and dynamic mechanical properties of PEMs and PECs. In turn, these approaches have been used to reveal fundamental connections relating the PEM/PEC structure to the glass transition and dynamic relaxation. Other excellent reviews cover the structure, properties, and applications of PEMs and PECs, to which we refer the reader.^51−56^

## Role of Hydration, pH, and Salt in the Glass Transition of Polyelectrolyte Multilayers/Complexes (PEMs/PECs)

One of our earliest (2012–2014) attempts to isolate the glass transition of a PEM used the quartz crystal microbalance with dissipation (QCM-D), in which the frequency and dissipation responses of PEMs adsorbed onto a quartz crystal sensor were monitored with temperature.^57−60^ However, we found that the technique’s results were inconsistent due to bubble formation in the instrument at higher temperatures and QCM-D lacks hydration control because the film is fully immersed. Our early work also considered PEM thickness and confinement effects, nanotube morphologies, and the processing method.^61−65^ By 2015, we turned to modulated differential scanning calorimetry (MDSC), which uses temperature modulation to enhance signals attributed to the glass transition, measured with a 2 °C/min rate in two heating/cooling cycles. Our MDSC work showed that the glass-transition temperature of a PEM or PEC decreased with increasing hydration.^66−71^ With the goal of connecting the glass-transition temperature to the physical structure of the PEM or PEC, neutron activation analysis (NAA) was also applied to determine the salt doping level and the water-to-intrinsic ion pair ratio, providing a connection of the observed thermal response to a PEM or PEC at the molecular level. Taken together, these methods allowed comparisons of glass-transition behavior between strong and weak polyelectrolyte systems, as shown in the following sections.

### Glass Transition in PEMs/PECs Containing Strong Polyelectrolytes

**Figure 1:**
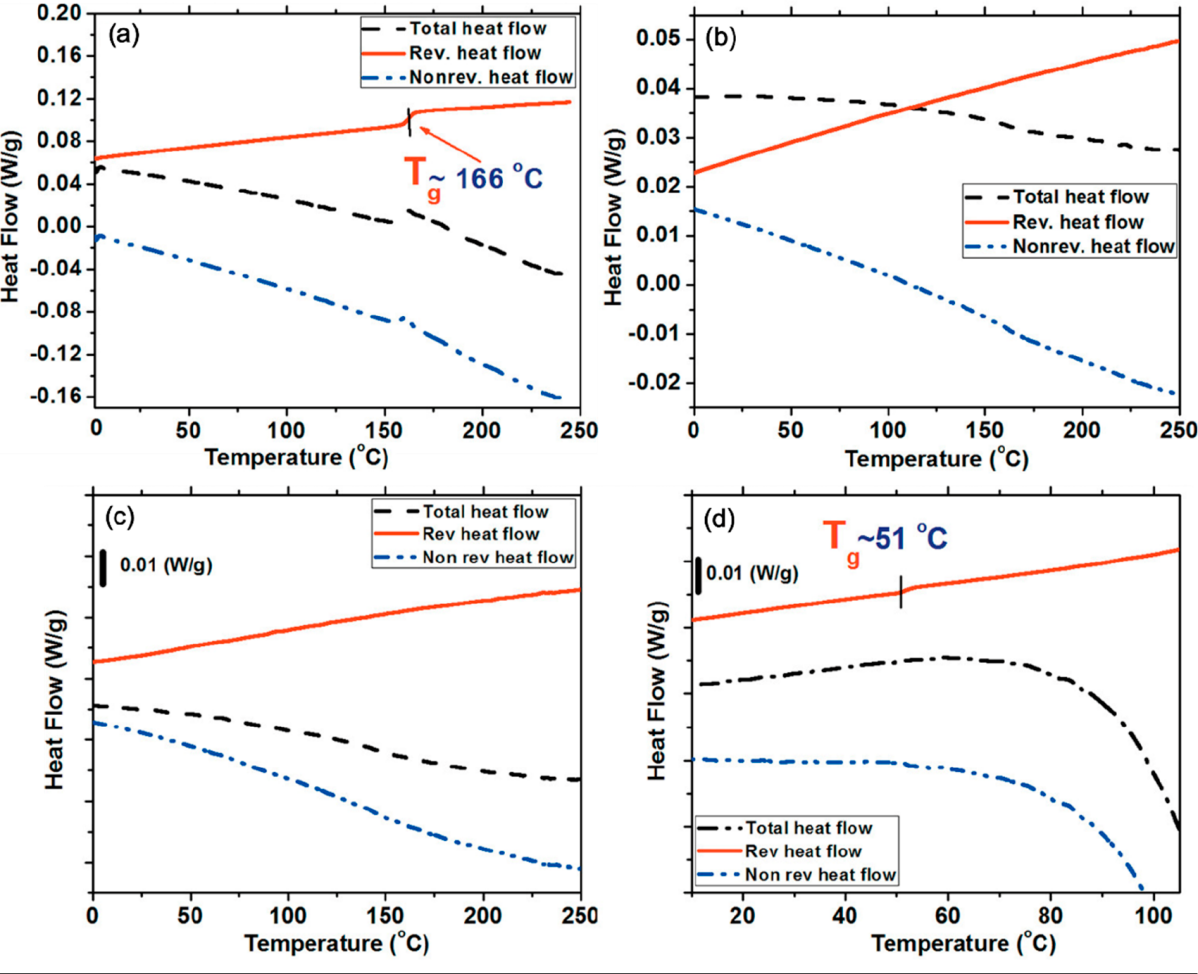
Modulated differential scanning calorimetry (MDSC) thermograms of (a) dry PDADMA and (b) dry PSS homopolymers. Ramped at 3 °C/min, amplitude of 1 °C, and period of 60 s. MDSC thermograms of (c) dry (0 wt % added water) and (d) hydrated (12 wt % added water) PDADMA/PSS LbL films assembled from 1.0 M NaCl. The cooling rate is 2 °C/min, amplitude of 1.272 °C, for 60 s. All presented MDSC thermograms represent the second heating scan. Reprinted (adapted) with permission from ref (57). Copyright 2012 American Chemical Society.

In 2012, we compared the glass transitions of dry homopolymers to those of their dry and hydrated multilayers.^57^ Strong polyelectrolytes poly(diallyldimethylammonium) (PDADMA) and poly(sodium 4-styrenesulfonate) (PSS) were specifically examined due to their extensive study in the PEM and layer-by-layer (LbL) communities. As strong polyelectrolytes, they remain ionized over a wide range of pH; therefore, their assembly behavior is expected to be influenced by salt rather than acidic or basic environments. Figure 1a and b shows the second MDSC heating cycle for dry PDADMA and PSS homopolymers, respectively. The inflection point in the reversing heat flow curve shows that dry PDADMA exhibited a *T*_g_ at 166 °C (Figure 1a) but dry PSS did not exhibit a *T*_g_ in the temperature range investigated (Figure 1b). Then, LbL films of PDADMA and PSS were assembled at varying ionic strengths. The LbL films were then dried in ambient air and stored in a desiccator until further use. But, interestingly, none of the dry LbL films had a detectable *T*_g_ (Figure 1c). The dry film’s glassy behavior was attributed to strong ion pairing between the quaternary ammonium and sulfonate groups. In contrast to the dry LbL films, hydrated LbL films demonstrated a glass transition. Herein, the total heat flow can be separated into reversing heat flow (which contains information regarding the heat capacity and *T*_g_) and nonreversing heat flow (which reflects enthalpy changes and kinetic events such as crystallization and decomposition). Specifically, Figure 1d shows an example of an MDSC thermogram for a hydrated (12 wt % water) PDADMA/PSS LbL film assembled from 1.0 M NaCl in which a thermal transition at 51 °C was observed in the reversing curve. This result was meaningful at the time because it clearly demonstrated the effect of water on the glass transition in multilayers.

**Figure 2:**
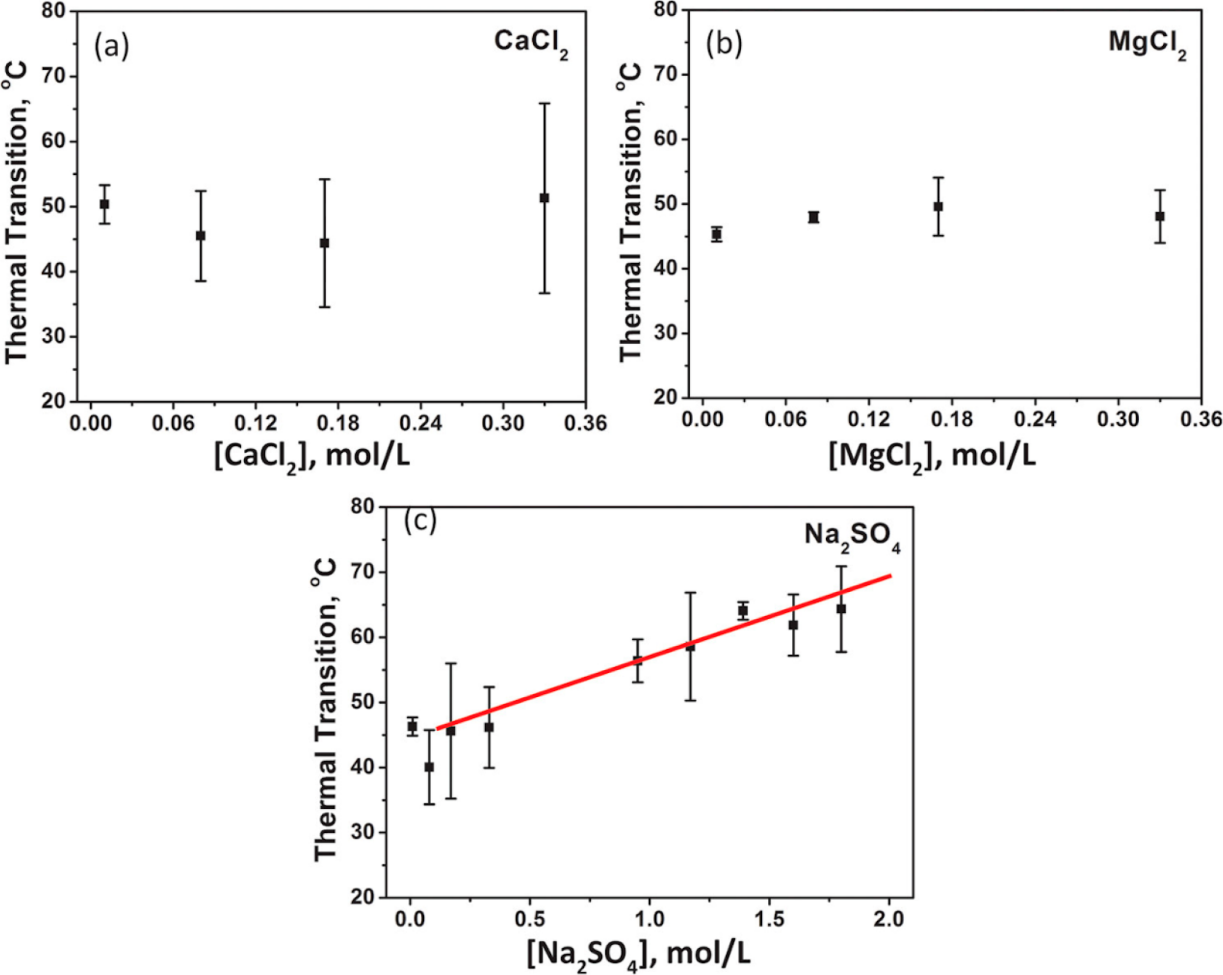
Concentration dependence of the thermal transition of PDADMA/PSS LbL films assembled from 0.5 M NaCl in contact with (a) CaCl_2_, (b) MgCl_2_, and (c) Na_2_SO_4_ solutions. The solid line is drawn to help guide the eye and represents a linear fit of *T*_tr_ = 12.4 *C*_Na_2_SO_4__ + 43.5 (*R*^2^ = 0.95). The error bars represent the standard deviation over at least three samples. Reprinted (adapted) with permission from ref (68). Copyright 2016 American Chemical Society.

With the prior study^57^ investigating only monovalent salts, we next sought to understand whether divalent cations and anions would affect the glass transition.^68^ PDADMA/PSS LbL assemblies were assembled in NaCl, isolated from the assembly substrate, and then exposed to solutions of either CaCl_2_, MgCl_2_, or Na_2_SO_4_. Figure 2 shows that the *T*_g_ (previously referred to as a thermal transition, *T*_tr_) increased with increasing Na_2_SO_4_ concentration but remained within error for CaCl_2_ or MgCl_2_. We note the relatively large error in *T*_g_ for the multilayers exposed to CaCl_2_ or MgCl_2_ at high concentration, which could arise from how the multilayers were prepared or exposed to the solutions. As for the effects on swelling, the multilayer thickness changed linearly with CaCl_2_ and MgCl_2_ concentration, but there was no remarkable trend in swelling as the Na_2_SO_4_ concentration increased.

In addition, O’Neal et al.^69^ investigated the glass-transition PDADMA/PSS multilayers by comparing the counterions in the system. Specifically, PDADMA/PSS multilayers were prepared in 0.5 M NaCl solutions and then subjected to different salt solutions (NaCl–KBr mixtures and KBr) postassembly at varying hydration levels (16–33 wt %). The results demonstrated that the hydration level influenced the *T*_g_ more so than the salt concentration. However, the salt type for assembly could strongly influence the *T*_g_, in which changing the assembly salt conditions from NaCl to KBr depressed the *T*_g_ by ∼20 °C. Additionally, compositional analysis based on nuclear magnetic resonance (^1^H NMR) spectroscopy and NAA revealed a direct relationship between the intrinsic and extrinsic ion pairing and the thermal transition of the polyelectrolyte LbL assemblies. The roles of water and salt were explained by molecular dynamics (MD) simulations of PDADMA/PSS assemblies.^70,71^ In particular, water has a plasticizing effect but salt can have a dual role contributing to a decrease or increase in plasticization via influencing the water structure in the assembly and breaking intrinsic ion pairs.^71^

**Figure 3:**
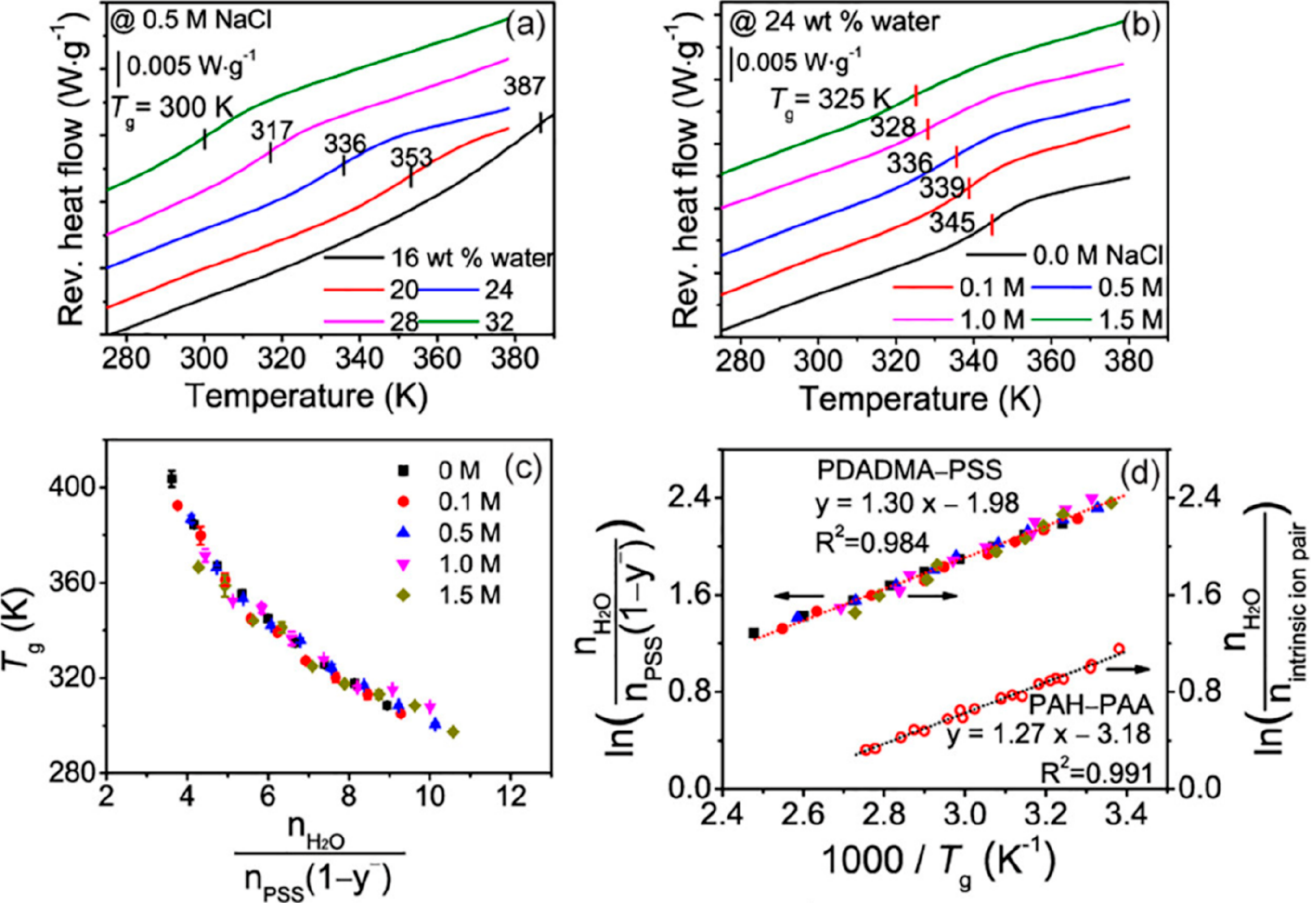
Reversing heat flow curves of modulated differential scanning calorimetry (MDSC) for PDADMA/PSS PECs of (a) varying water content and fixed 0.5 M NaCl complexation concentration and (b) varying NaCl complexation concentration and fixed water content of 24 wt %. (c) *T*_g_ as a function of the molar ratio of water molecules to the intrinsic ion pair in hydrated PDADMA/PSS complexes prepared from solutions of different NaCl concentrations. (d) Linear fitting of ln(*n*_H_2_O_/*n*_intrinsic ion pair_) vs 1000/*T*_g_ (dotted lines). For (a) and (b), the MDSC second heating scans are shown with “exotherm down”, a heating rate of 2 K·min^–1^, and an amplitude of 1.272 K for a period of 60 s. The legend in (c) also applies to (d). The left *y* axis applies to PDADMA/PSS, and the right *y* axis applies to both PDADMA/PSS and PAH/PAA. PAH/PAA data are from the authors’ previous work.^67^ Reprinted (adapted) with permission from ref (66). Copyright 2018 American Chemical Society.

Building upon this prior work, we reported in 2018 a scaling for water, intrinsic ion pairing, and the glass transition that described both PEMs and PECs as well as PECs made from both strong and weak polyelectrolytes.^66^ The glass-transition temperatures of PDADMA/PSS complexes prepared at varying salt concentration (0–1.5 M NaCl) and exposed to different hydration levels (16–32 wt %) were quantified. For a fixed complexation salt concentration of 0.5 M, the *T*_g_ decreased from 387 to 300 K as the water content increased from 16 to 32 wt % (Figure 3a). Figure 3b shows the response for PECs of varying complexation NaCl concentrations at a fixed hydration level of 24 wt %; the *T*_g_ decreased from 345 to 325 K as the NaCl concentration increased from 0 to 1.5 M, indicative of a salt plasticization effect.

To explore water–salt–temperature relationships for the PDADMA/PSS system, the *T*_g_ values were plotted against the ratio of water molecules to intrinsic ion pairs (), shown in Figure 3c. The ratio can also be expressed in terms of the cation or anion doping level  or , depending on which species is a minority. Here, the molar ratio of water to intrinsic ion pairs becomes  because PSS was the minority component, where *n*_PSS_ is the number of PSS monomers. NAA was used to estimate *y*^+^ and *y*^–^, and the water content *n*_H_2_O_ was taken as the controlled amount of water added to the system. Also, *n*_intrinsic ion pair_, which is defined as the number of intrinsic ion pairs (polycation–polyanion pairs), was estimated from a charge balance, a mole balance, and the doping level. Remarkably, the *T*_g_ values all collapsed into a single master curve for all hydration levels and salt concentrations examined. To make a link between the glass transition and water content in the PECs, the linearization of this master curve, shown in Figure 3d, yielded the following scaling:
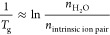
1The successful collapse and linearization of *T*_g_ values for PDADMA/PSS complexes is notably similar to that observed in poly(allylamine hydrochloride)/poly(acrylic acid) (PAH/PAA) complexes.^67^ The slope corresponding to eq 1 in Figure 3d allows for calculating an energy, presuming the relationship follows a van’t Hoff-like equation. Remarkably, the van’t Hoff enthalpy measured for the PDADMA/PSS complexes was nearly identical to that of PAH/PAA complexes, −10.8 vs −10.5 kJ·mol^–1^, respectively. These values are close to the van’t Hoff enthalpy of hydrogen bond disruption between two water molecules,^72^ which further suggests the generalized role of water in the relaxation. However, the *y* intercepts for the linear fits were different for the different PEC systems, which we attribute to differences in the charged group size or ion pair size (e.g., the PDADMA repeat unit is larger than the PAH repeat unit), the linear charge density, and/or the water distribution around the charged groups between these two PEC systems. Notably, the aforementioned characteristics of the system may alter entropic contributions. In addition, MD simulations for the PDADMA/PSS complex also show a relationship between *T*_g_ and the hydrogen-bonding environment, further supporting the contribution of water in weakening intrinsic ion pairing and promoting the sliding motion of polyelectrolytes.^66^

**Figure 4:**
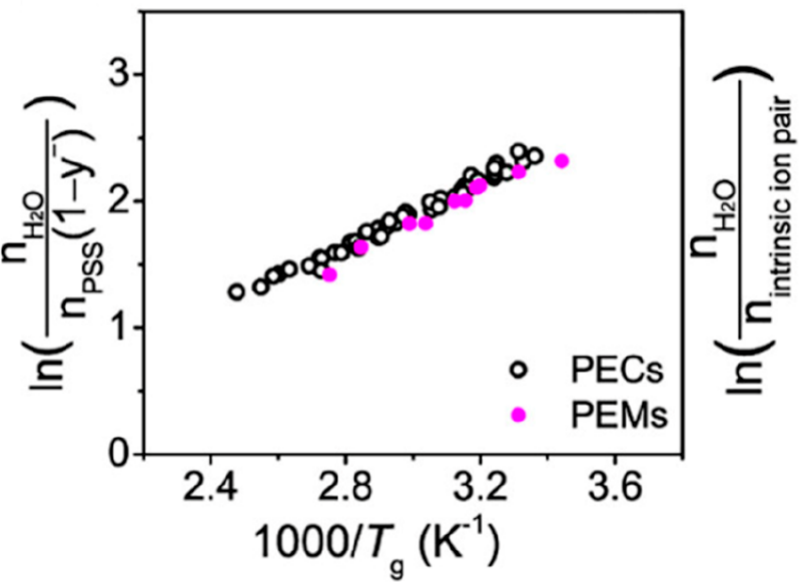
Plot of ln(*n*_H_2_O_/*n*_intrinsic ion pair_) vs 1000/*T*_g_ for PDADMA/PSS polyelectrolyte complexes (PECs, black circles) and polyelectrolyte multilayers (PEMs, pink circles). Reprinted (adapted) with permission from ref (66). Copyright 2018 American Chemical Society.

The generalized scaling of *T*_g_ for PECs with water at the intrinsic ion pairs is useful for relating the molecular-level internal structure to polymer relaxation, but the question of if this behavior was similar for charged assemblies made by a different path remained. To examine this, we performed the same analysis for PDADMA/PSS PEMs assembled using the LbL technique.^66^ In Figure 4, the relationship between *T*_g_ and the molar ratio of water to the number of intrinsic ion pairs shows a collapse of all of the measured *T*_g_ values into a single linear master curve for both PECs and PEMs. The results from this study are remarkable as they highlight a potentially generalized phenomenon for the relaxation of macromolecular assemblies with charged ion pairs.

In parallel with experiment, we sought to investigate the molecular contributions of water to explain the PEC’s behavior. Specifically, the state of water (bound vs nonbound) and its diffusion (rotational and translational) in the PEC may influence the *T*_g_. Batys et al.^73^ investigated the dynamics of water mobility in hydrated PDADMA/PSS complexes and its reliance on the temperature and hydration level using MD simulations. The results showed that at 26 wt % water content only very strongly bound water was present in the PECs, implying a strong PE–water association. We have confirmed this by DSC measurements, which indicated only the presence of nonfreezing water in that hydration range.^73^ The water diffusion coefficients that were estimated from MD simulations for PECs at high hydration levels and elevated temperatures exhibited strong contributions from translational motion, whereas rotational motion dominated water dynamics at low temperatures and hydration levels.^73^ Increases in either PEC hydration or temperature led to increased water mobility, providing a lubrication effect. Taken together, MD simulations validated the roles of temperature and hydration in plasticization of the PEC.

### Glass Transition in PEMs/PECs Containing Weak Polyelectrolytes

In contrast to the PDADMAC/PSS system, which consists of strong polyelectrolytes with degrees of ionization that are independent of pH, PECs and PEMs consisting of weak polyelectrolytes have degrees of ionization that are highly sensitive to pH. To understand the influence of pH, we have studied PECs and PEMs composed of PAH and PAA, which are weak polyelectrolytes with p*K*_a_ values of 8–10 and 5.7–6.5,^58,67,74^ respectively. For example, if the pH value is below the p*K*_a_ of PAA, then PAA will favor protonation rather than ionization; conversely, for PAH, ionization would be favored. As a consequence, the pH value will influence the number of intrinsic ion pairs, extrinsic ion pairs, and protonated units and therefore the thermal properties of the PAH/PAA system.

**Figure 5:**
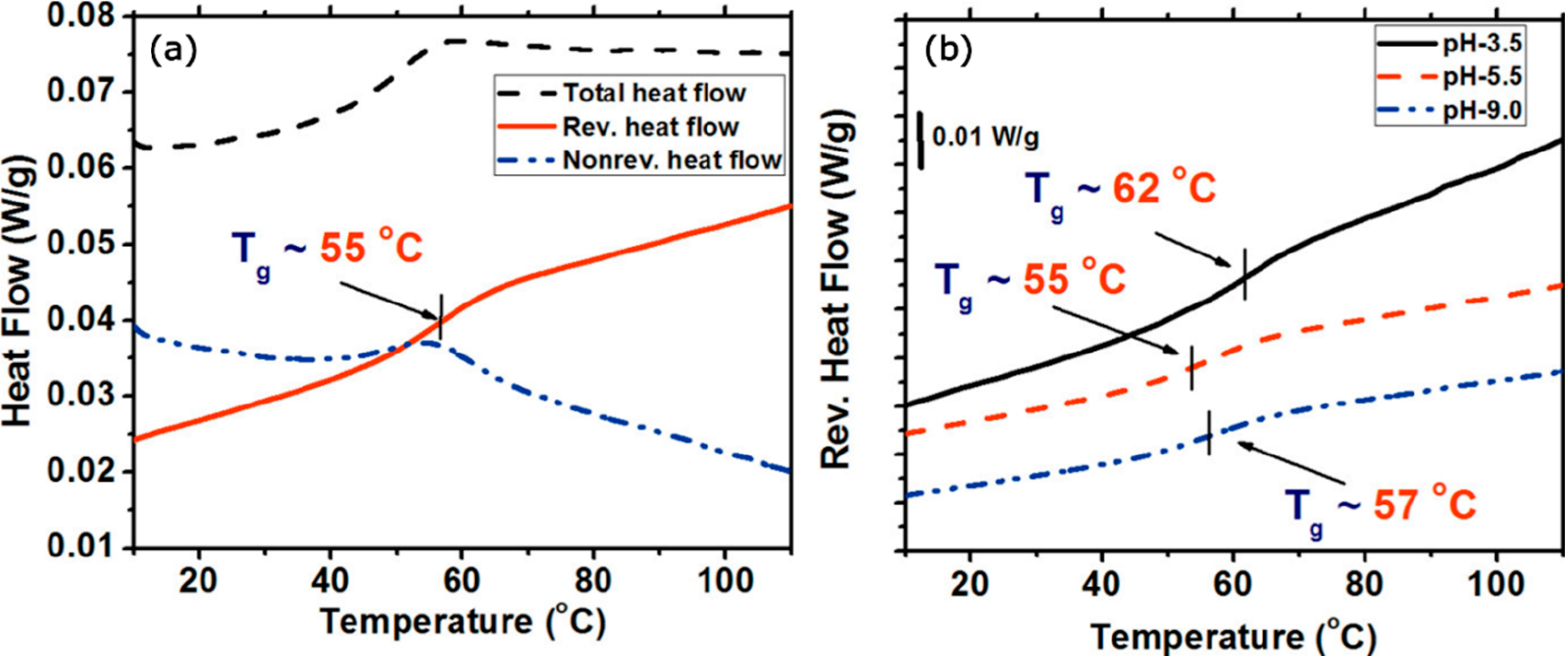
(a) MDSC thermograms of hydrated PAH/PAA LbL films assembled from pH 5.5 solution. (b) Reversing heat flow curves for hydrated PAH/PAA LbL films assembled from pH 3.5, 5.5, and 9.0 solutions. Curves in (b) have been shifted along the *y* axis for clarity. Heating rate of 2 °C min^–1^, amplitude of 1.272 °C, and period of 60 s (second heating cycle shown). Reprinted (adapted) with permission from ref (58). Copyright 2012 American Chemical Society.

In 2010, we investigated the thermochemical properties of dry PAH/PAA LbL assemblies.^75^ Dry PAA and PAH homopolymers exhibited *T*_g_’s of 128 and 223 °C, respectively, but a dry PAH/PAA LbL film exhibited no evidence of a glass transition. Instead, cross-linking via amidation was observed. Two years later, we investigated pH effects upon the glass transition of hydrated LbL films using MDSC (Figure 5).^58^ Notably, the hydrated PAH/PAA LbL films exhibited distinct *T*_g_’s that varied with assembly pH. As shown in Figure 5a, a distinct glass transition at 55 °C was observed in the reversing curve, accompanied by enthalpic relaxation associated with physical aging observed in the nonreversing curve for a PAH/PAA LbL film assembled at pH 5.5. Figure 5b shows the reversing heat flow curves for hydrated (18 wt % water) PAH/PAA LbL films assembled from various pH-adjusted solutions. For assembly pH values of 3.5, 5.5, and 9.0, the *T*_g_’s were 62, 55, and 57 °C, respectively.

**Figure 6:**
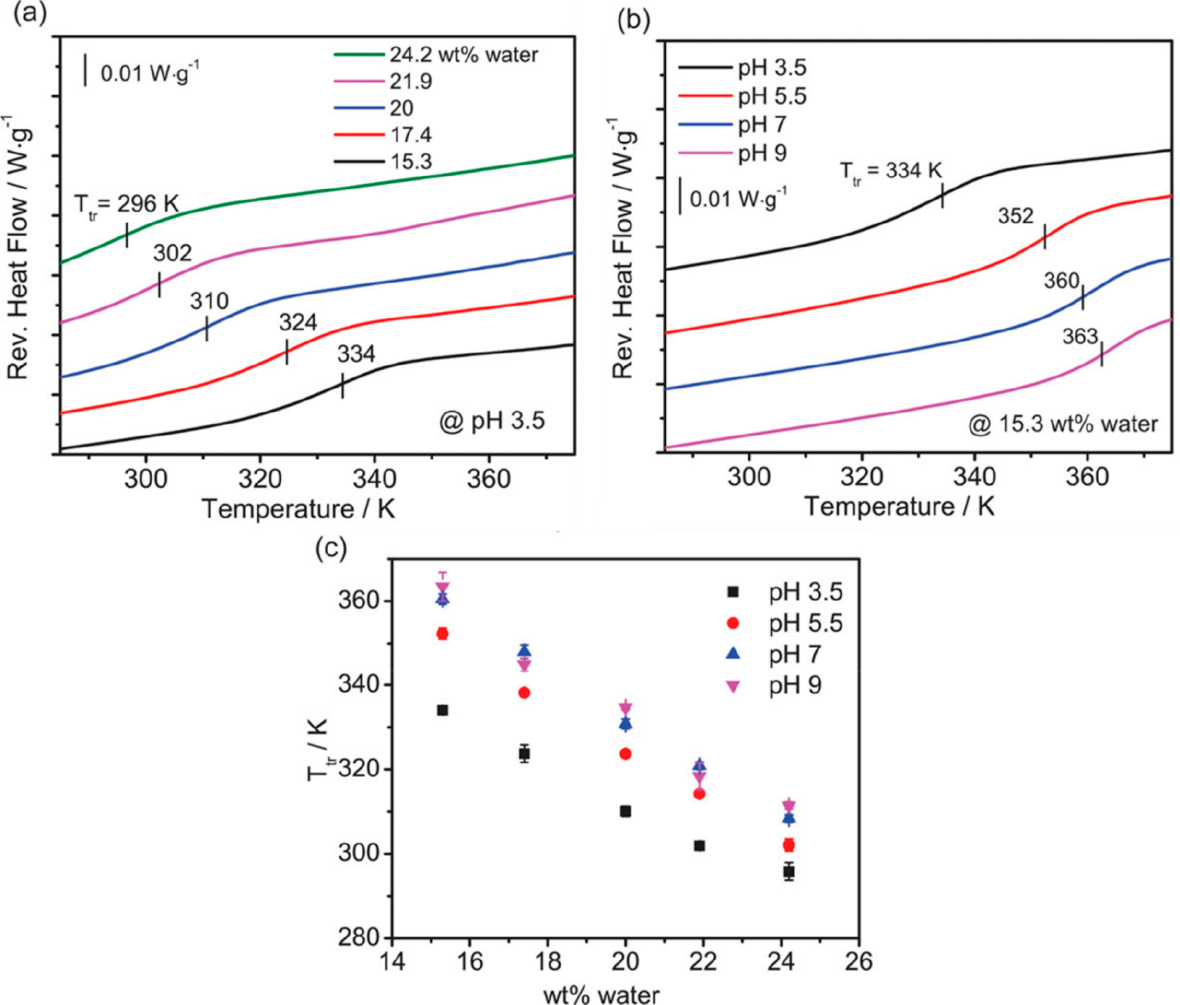
Reversing heat flow curves of modulated differential scanning calorimetry (MDSC) of (a) (PAH/PAA)_3.5_ complexes of varying water content and of (b) varying complexation pH values and constant water content (15.3 wt % water). For (a) and (b), second heating scans are shown, and curves have been shifted along the *y* axis for clarity. (c) *T*_tr_ for PAH/PAA complexes for varying complexation pH values and water content. Reprinted (adapted) with permission from ref (67). Copyright 2016 American Chemical Society.

**Figure 7:**
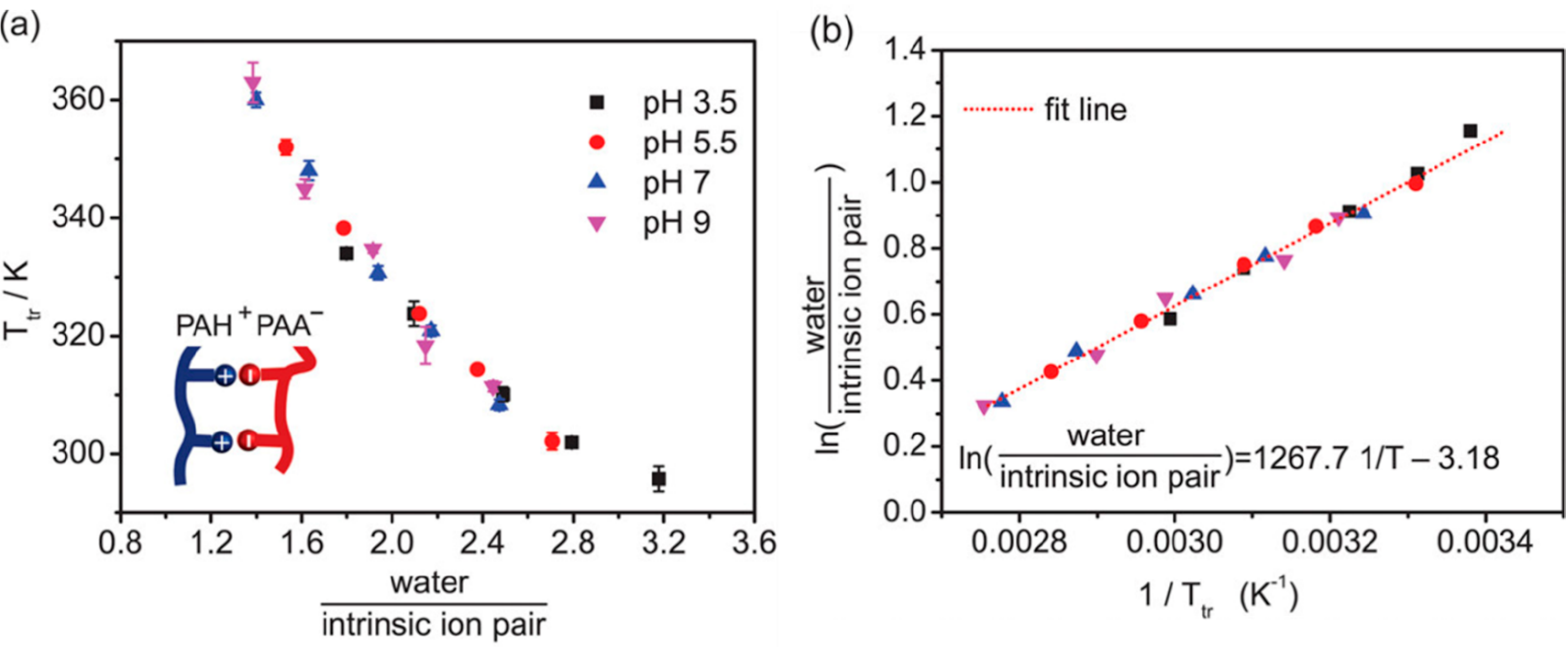
(a) *T*_tr_ vs number of water molecules per intrinsic ion pair in hydrated PAH/PAA PECs prepared at pH 3.5, 5.5, 7, and 9. The number of water molecules is taken as the total amount water added to the complex. The number of intrinsic ion pairs is calculated from the PEC mass and PAH/PAA composition, assuming that all PAH units are ionized and participate in intrinsic ion pairing. (b) Linear fitting of ln(water/intrinsic ion pair) vs 1/*T*_tr_. The legend in (a) also applies to (b). Reprinted (adapted) with permission from ref (67). Copyright 2016 American Chemical Society.

The origin of the variation of *T*_g_ with assembly pH for the PAH/PAA LbL films was not well understood at the time of ref (58), motivating a deeper investigation of analogous PAH/PAA complexes. Using MDSC, the *T*_g_ was identified for PAH/PAA complexes prepared at different pH values and examined under varying levels of hydration. Figure 6a shows that the thermal transition temperature (*T*_tr_, which is equivalent to the *T*_g_) of a PAH/PAA PEC prepared at pH 3.5 decreased with increasing hydration. This shows that the presence of water strongly affects the thermal transition. Notably, the plasticizing effect of water decreased the *T*_tr_ as hydration increased. Figure 6b shows that the *T*_tr_ (or *T*_g_) increases with increasing complexation pH for PAH/PAA PECs of a constant hydration level (15.3 wt %). Figure 6c summarizes all of the obtained *T*_tr_ values for the various pH values and hydrations. Given the similar trends in *T*_tr_ with respect to water wt % in the system shown in Figure 6c, we desired to formulate a more universal representation of the response. This resulted in Figure 7a, in which a plot of *T*_tr_ versus the number of water molecules divided by the number of intrinsic ion pairs resulted in a single master curve, confirming a direct relationship among the three parameters. Furthermore, a plot of the log of the ratio of water molecules per intrinsic ion pair vs the inverse of the transition temperature showed a collapse of the data into a single, linear master curve (Figure 7b). Shown below, the linear fit was described from physical principles in eqs 2 to 11 for which *T* can represent *T*_g_ or *T*_tr_.

The successful collapse of *T*_tr_ into a single master curve without any adjustable parameters is remarkable, especially considering that this had not been previously demonstrated. The generalized master curve response implies that water associated with intrinsic ion pairs plays a role in the thermal relaxation, consistent with the discussion above centering around ref (66). Specifically, the linearization of *T*_g_ for the PAH/PAA complexes was comparable to that of the PDADMA/PSS PECs (see Figure 3d), where the two systems exhibited similar slopes.^66^ Taken together, this suggests that the glass transition of a PEC occurs by a water-mediated relaxation at the intrinsic ion pair, where water plasticizes and lubricates the system. In support of this, our MD simulations^70^ on PDADMA/PSS systems suggested that the thermal transition results from the rearrangement and dynamic changes of water molecules around the polyanion.

### Speculation on the Physical Interpretation of the Glass Transition’s Relationship to Water and Intrinsic Ion Pairing

The scaling of *T*_g_ with the ratio of water molecules to intrinsic ion pairs presented in eq 1 was, when first presented in 2016,^67^ phenomenological. With further study of two different systems (PDADMA/PSS and PAH/PAA) and two different processing approaches (PEMs and PECs) under different pH values, ionic strengths, and assembly salts, the scaling presented within eq 1 remained valid, as described in the two prior sections. Here, we present a speculative description on a possible physical interpretation that captures the essence of eq 1.

Consider an intrinsic ion pair with a surrounding shell of water molecules. In one extreme, this intrinsic ion pair may exist as a contact ion pair in which the positively and negatively charged PE groups do not have any solvent molecules between them. Solvation of the PE–PE ion pair can involve water molecules between the PE–PE ion pair as part of the hydration shell of the intrinsic ion pair, creating a solvent-separated ion pair. Such solvent-separated polycation–polyanion pairs are weaker because the water molecules are electric dipoles, locally screening the PE ionic group charges.^76^ The more water molecules in the solvation shell of the PE–PE ion pair, the more efficiently screened is the intrinsic ion pair. This proposed mechanism allows the formulation of an equilibrium between an intrinsic ion pair (IP) hydrated with *n* water molecules and an IP that is hydrated with *n* + 1 water molecules:
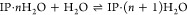
2The insertion of additional water into the hydration shell of the intrinsic ion pair may also be sufficient to allow further relaxation of the assembly via the intrinsic ion pair either rearranging with another adjacent ion pair or by allowing it to break to form an extrinsic ion pair. Our molecular dynamics simulations indicate that hydration does not strongly affect the fraction of intrinsic/extrinsic ion pairing. For some PE pairs, such as PDADMA/PAA, the increase in hydration slightly decreases intrinsic ion pairing, and slight changes in intrinsic ion pair number with relative humidity have also been observed for PAH/PAA systems.^76,77^ However, the decrease in intrinsic ion pairs remains minor in comparison to the change in their dynamics. Presuming that rearrangement of a weakened intrinsic ion pair primarily occurs via exchange with adjacent intrinsic ion pairs, we propose that intrinsic ion pairs (with or without solvent separation) may experience transition states where increasing hydration induces brief separation but the ion pair remains positionally correlated
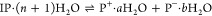
3where P^+^·*a*H_2_O represents a polycation repeat unit hydrated by *a* water molecules and P^–^·*b*H_2_O represents a polyanion repeat unit hydrated by *b* water molecules. The moles of water in the complex is related to the coefficients as *n* + 1 = *a* + *b*. The addition of the two reactions yields
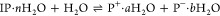
4where the equilibrium between the intrinsic ion pair’s states is represented by
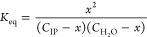
5where *x* represents the change in concentration of cation/anion pairs due to the conversion of contact intrinsic ion pairs into a solvent-separated state (or a solvent-separated pair into one with an additional water molecule included in its hydration shell). We propose that there exists some critical fraction of sufficiently solvent-separated (or extra-hydrated) intrinsic ion pairs, *y*, for which a bulk-scale relaxation is permitted, which may be represented as eq 6, yielding a new expression for the equilibrium as eq 7:
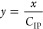
6
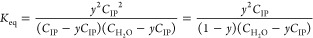
7Presuming that only a small fraction of solvent-separated (or extra-hydrated) intrinsic ion pairs are required to undergo a relaxation such that *yC*_IP_*≪ C*_H_2_O_, eq 7 becomes
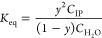
8Finally, the equilibrium can be related to the reaction’s enthalpy and entropy by the van’t Hoff relationship
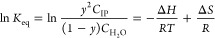
9and
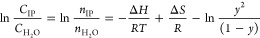
10
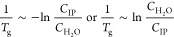
11where Δ*H* and Δ*S* represent the respective enthalpy and entropy associated with the conversion of the intrinsic ion pair from a tightly bound ion pair to a more solvent-separated ion pair. If we presume that a *T*_g_-like event occurs when there exists a sufficient fraction of solvent-separated ion pairs *y*, then we retrieve a scaling similar to that presented in eq 1.

By this reasoning, we revisited our data from prior publications and replotted them in the form of  vs  (Figure S1). The slope remains consistent with the van’t Hoff enthalpy of a water hydrogen bond disruption (−10.5 kJ/mol) as before.^66,67^ The PDADMA/PSS and PAH/PAA systems have different *y* intercepts, which may be interpreted as entropic differences and/or differences in the critical value of *y* to undergo the relaxation.

We acknowledge that this treatment is speculative because we do not have direct evidence of the extent of contact vs solvent-separated ion pairs. This explanation also does not directly incorporate extrinsic ion pairing, but we have found that, through our studies of PAH/PAA, extrinsic ion pairing is indirectly included in that it affects *C*_IP_ and *n*_IP_. And as shown in Figure 3(b), the addition of NaCl could decrease *T*_g_ due to the breakup of intrinsic ion pairs due to screening.

## Influence of Water on the Dynamics of PECs and PEMs

PECs can show multiple relaxation times (τ), with shorter time scale relaxations attributed to the breaking and reformation of intrinsic ion pairs and longer time scale relaxations corresponding to the relaxation of polyelectrolyte chains. The dynamics at the intrinsic ion pair are closely related to ionic strength, which, as shown above, is linked to the glass transition by the salts’ ability to change the number of intrinsic ion pairs. In turn, salt can strongly affect the mechanical properties of PECs and PEMs.^56^ For example, Spruijt et al. found that the storage and loss moduli of PECs decrease with increasing salt concentration, resulting from the added salt decreasing the number of intrinsic ion pair cross-links.^78^ The dynamics of the PEC were described using time–salt superpositioning, indicating that the salt concentration did not alter the PEC’s relaxation mechanism. However, PECs and PEMs dissolve at sufficiently high salt concentrations.^57,79^ Elsewhere, Akkaoui et al. found that the long-term relaxation time of coacervates decreased with increasing salt concentration.^80^ We also pointed out by MD simulations the dual role of adding salt.^71^ Taken together, the dependence of mechanical properties and relaxation times on salt concentration has been studied extensively. However, the impact of water is less understood. In our group, we have studied the dependence of water on the mechanical properties and relaxation of PAH–PAA PECs, including time–water, time–water–temperature, and time–water–temperature–pH relationships, to reveal a scaling relationship that connects relaxation time to water and intrinsic ion pairing.

### Effect of Water on the Ionic Conductivity, Mechanical Properties, and Chain Mobility of PECs and PEMs

Other groups have observed the strong influence of water in the properties of PEMs and PECs. For example, water enhances the conductivity of PECs and PEMs,^81−85^ in which ionic transport is described by the dynamic structure model (DSM). In DSM, ionic transport occurs via an ion hopping mechanism in which the polyelectrolytes are undergoing local rearrangement.^85^ For example, Akgol et al. observed that the DC conductivity, σ_DC_, of PEMs increased with relative humidity (RH) for a range of polyelectrolyte systems by the following equation^81^
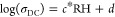
12where *c* and *d* are fitting constants. Later, De et al. attributed the enhanced ionic mobility to a reduction in the activation energy for ion transport caused by the increased volume fraction of water.^84^ Cramer et al. studied the conductivity of PDADMA/PSS PECs at different RH values, leading to the successful application of time–humidity superpositioning, proving that the ion transport mechanism does not change with the PECs’ water content.^83^

Water has also been shown to plasticize and soften PEMs and PECs. For example, Wang et al. studied the effect of RH on chain mobility in polyelectrolyte complex nanoparticles using fluorescence recovery after photobleaching (FRAP), in which humidity annealing promoted recovery.^86^ Elsewhere, Huang et al.^87^ studied the mechanical properties of polyelectrolyte complex fibers of alginate and PDADMA as a function of RH; the initial modulus and ultimate tensile strength continuously decreased whereas the breaking strain increased with increasing RH. In a different study, Hariri et al.^50^ studied the mechanical properties of PDADMA/PSS PEMs as a function of water content using nanoindentation in which the elastic modulus increased as the water content decreased by the following equation
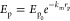
13where *E*_p_ is the elastic modulus of the plasticized PEM, *E*_p_0__ is the elastic modulus of the dry PEM, *k*_m_ is the plasticizer efficiency, and *r*_p_ is the mole ratio of the plasticizer (i.e., water) to polymer. This further highlights that water acts as a plasticizer in PECs and promotes chain mobility.

### Effect of Water on Dynamic Mechanical Properties and Relaxation Time in PECs

**Figure 8:**
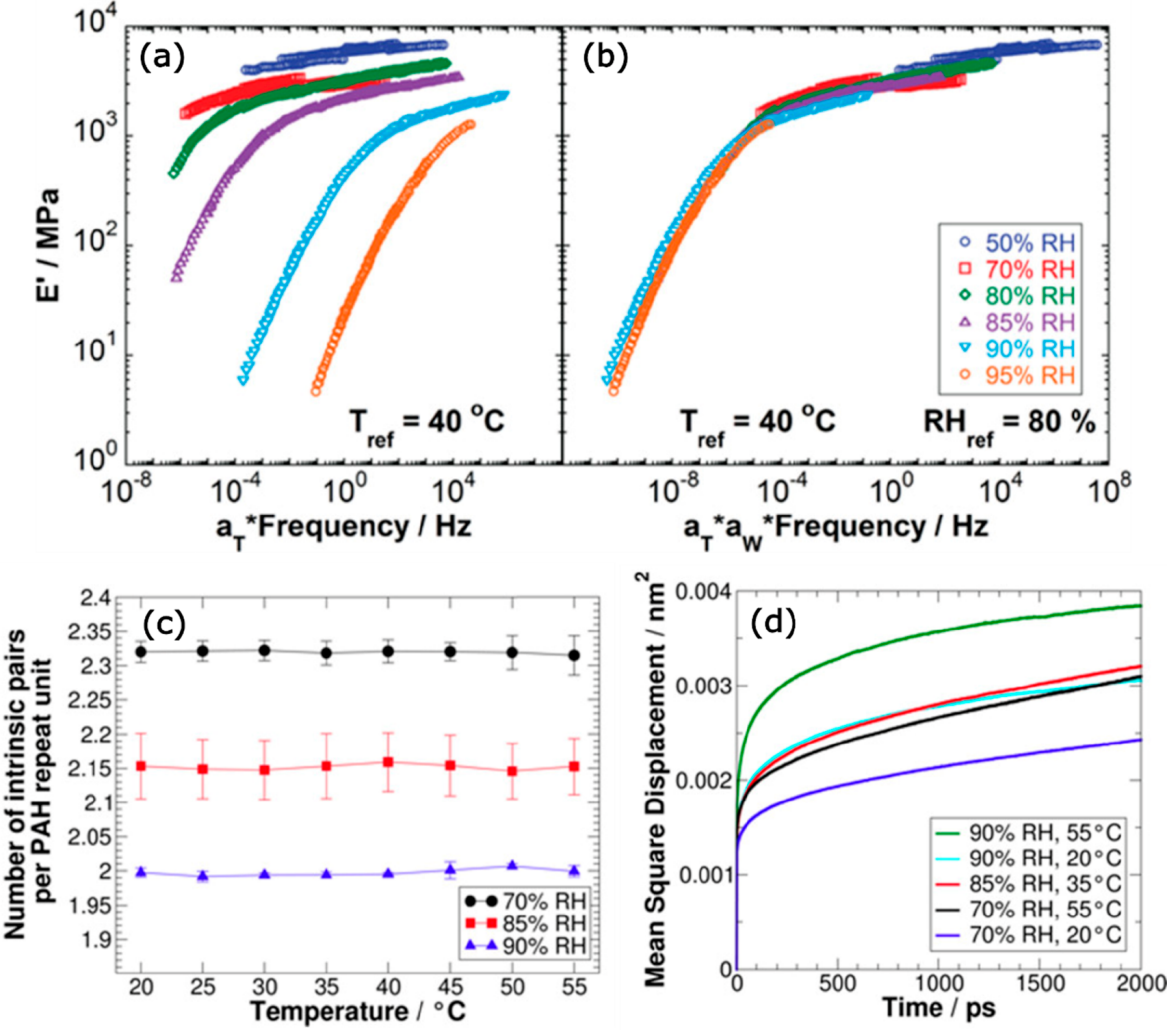
(a) Time–temperature master curves for RH values of 50, 70, 80, 85, 90, and 95% for a PAH/PAA PEC system. (b) Time–temperature–water super master curve compiled using time–temperature master curves in (a) with RH_ref_ = 80% and *T*_ref_ = 40 °C. The legend in (b) also applies to (a). (c) Number of intrinsic ion pairs per single PAH repeating unit as a function of the temperature and relative humidity from MD simulations of PAH/PAA PEC models at corresponding RHs. (d) Mean square displacement (MSD) of PAH chains at different hydration levels and temperatures in the MD simulations. Reprinted (adapted) with permission from ref (77). Copyright 2019 American Chemical Society.

Motivated by the time–humidity superposition principle mentioned earlier, we applied the principle of time–temperature–water superpositioning to the dynamic mechanical properties of PAH/PAA PECs prepared at pH 7.0.^77^ First, time–temperature superposition (TTS) was performed for dynamic mechanical data measured at different relative humidity values (50–95%). The horizontal temperature shift factor (*a_T_*) obtained after performing TTS at a given RH value was described by the Arrhenius equation. However, as the RH increased, the activation energy from the Arrhenius relationship for the *a_T_* values also increased. Figure 8a shows the TTS master curves obtained at different RH values. *E*′ (storage modulus) decreased with increasing RH due to softening of the PEC by water, which acts as a plasticizer. The TTS curves obtained at different RH values were superposed to obtain a time–temperature–water superposition (TTWS) master curve (Figure 8b). Interestingly, the shift factors corresponding to the TTWS, *a*_W_, followed a log–linear relation with respect to the PEC’s water content
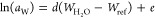
14where *W*_H_2_O_ is the weight % of water in the PEC, *W*_ref_ is the corresponding weight % of water at the chosen reference RH, *d* is the slope, and *e* is the *y* intercept. Interestingly, eq 14 mirrors eq 12, which describes the humidity dependence of conductivity in PECs and PEMs.

All-atom molecular dynamics simulations of the PAH/PAA PEC system were performed to understand if water content or hydration would affect the types of ion pairs present within the PEC.^77^Figure 8c shows that the number of intrinsic ion pairs remains unchanged within the studied temperature range and is only slightly affected by the different levels of hydration, which suggests that TTW is valid for the temperature and hydration range studied. Also, PAH chain mobility in the PECs was investigated for varying temperatures and hydration levels (Figure 8d). The PAH chain mobility was similar for 85% RH at 35 °C and 70% RH at 55 °C, which highlights that temperature and water show similar effects on the mean square displacement (MSD) of PAH chains. The same behavior was observed for PAA chains. As before with PDADMA/PSS PECs,^73^ both temperature and hydration result in a similar plasticization effect in the PEC, which explains why the temperature and hydration can be treated as mutually interchangeable factors.

**Figure 9:**
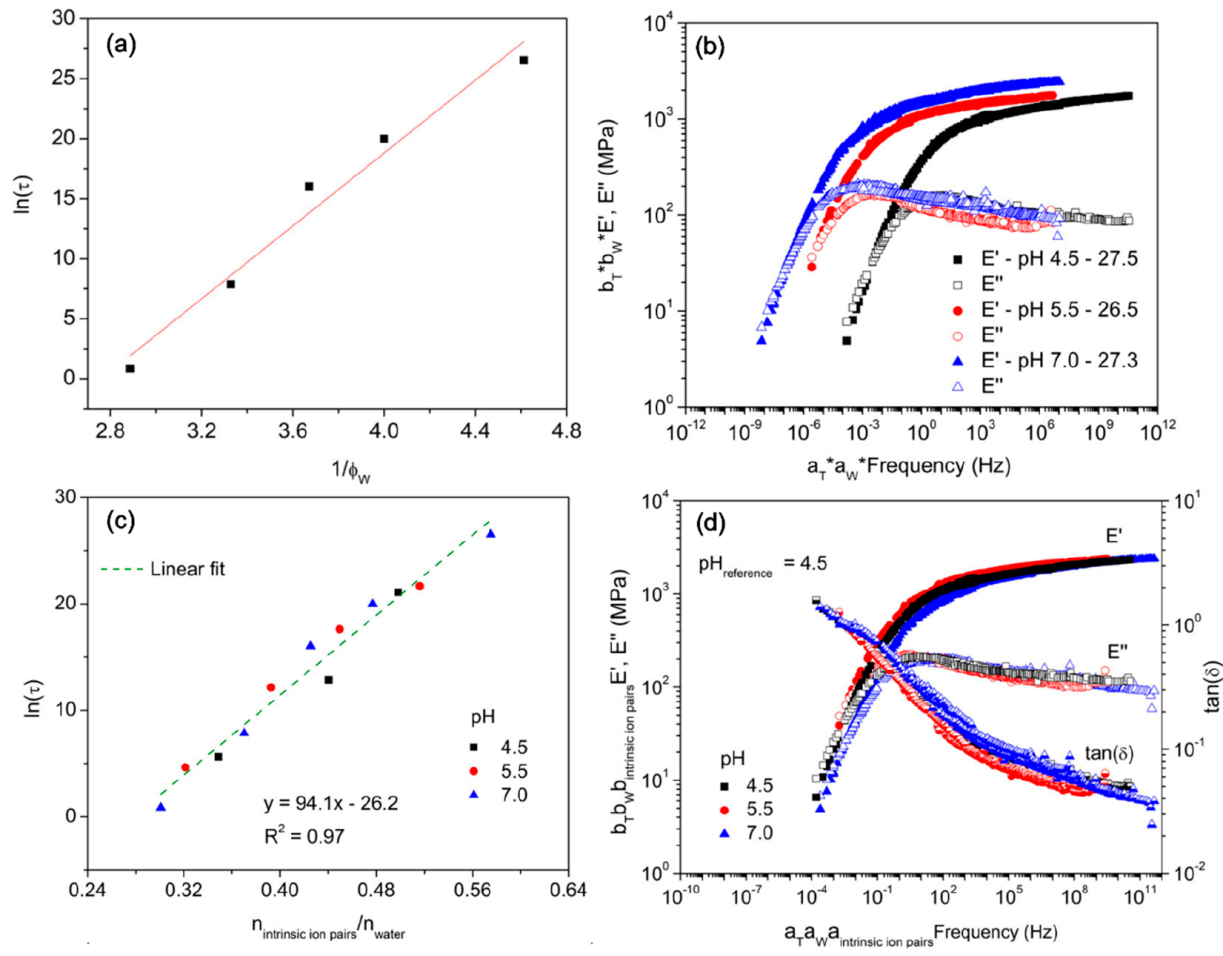
(a) Natural log of relaxation time vs the inverse of the volume fraction of water in PAH/PAA PECs. The solid line depicts the fit of a free volume model (). (b) Dynamic mechanical data (*E*′, storage modulus and *E*″, loss modulus) of PAH/PAA complexes at pH values of 4.5, 5.5, and 7.0 at an equivalent volume fraction of water (27%). The curves have been shifted using TTWS. (c) Natural log of relaxation time τ vs the ratio of the number of intrinsic ion pairs to the number of water molecules for PAH/PAA PECs at pH values of 4.5, 5.5, and 7.0. The green dashed line shows a linear fit of the data. The number of intrinsic ion pairs was calculated by assuming that PAH was fully ionized and that every PAH repeat unit formed an intrinsic ion pair. (d) Time–temperature–water–pH superposition with pH 4.5 as the reference pH, 30 °C as the reference temperature, and *W*_ref_ as the reference water percentage. Reprinted (adapted) with permission from ref (88). Copyright 2021 American Chemical Society.

The previous study was performed for PAH/PAA PECs at one complexation pH, i.e., pH = 7.0. In 2021, we extended our study to include multiple complexation pH’s.^88^ First, TTS was performed at different RH values for a particular pH of complexation. Next, time–temperature–water master curves were produced by performing TTWS. The master curves in this case showed an excellent overlay of tan(δ). The relaxation time was estimated by the inverse crossover frequency using the dynamic mechanical data and was found to be inversely proportional to the volume fraction of water, *ϕ*_W_) (Figure 9a). Doolittle^89^ studied the viscosity of entangled polymer systems and postulated that viscosity is inversely proportional to the free volume fraction, *ϕ*_f_. This suggests that, in the case of hydrated PECs, water acts as a plasticizer and provides free volume to facilitate chain relaxation. This is closely tied to the prior observations of increased ionic conductivity with increasing water content in PECs.^82^ As discussed below, the microenvironment of water in the PEC, whether it be bound or free, strongly influences the PEC’s relaxation.

Next in our efforts to resolve the effect of water on the mechanical properties of PECs, we moved to an analysis of the dynamic mechanical response of PAH–PAA complexed at different pH values (4.5, 5.5, and 7.0). As before, the TTWS approach was taken. As shown in Figure 9b. *E*′ decreased as the complexation pH decreased. This can be explained by a smaller number of intrinsic ion pairs with a smaller complexation pH. Additionally, the crossover frequency of *E*′ and *E′′* (loss modulus) shifted to higher frequency values with decreasing complexation pH (Figure 9b). This indicates that the PEC relaxation time is dependent on the complexation pH at similar volume fractions of water. Therefore, *E*′ and the relaxation time, τ, both decrease with decreasing complexation pH. Interestingly, a plot of ln(τ) versus the number of intrinsic ion pairs/number of water molecules led to a collapse of the relaxation times obtained for the different pH values examined (Figure 9c). In comparison, no such collapse was obtained for when ln(τ) was plotted against the inverse of the volume fraction of water. This supports a conclusion that the relaxation in hydrated PAH–PAA PECs was mediated by water molecules at the intrinsic ion pairs. This finding relates closely to previously reported studies from our group on the effect of water on the glass-transition temperature.^66,67^ Additionally, it attracts attention to the key role of bound water and the degree of water binding at the ion pairs, as the local hydration of the ion pair appears to be key to the response. Last, a Kohlrausch–Williams–Watts (KWW) model was used to validate that the dynamics were consistent across the conditions studied, which then allowed for the application of time–temperature–water–pH (or intrinsic ion pair) superpositioning (Figure 9d). Elsewhere, Tekaat et al. successfully applied time–pH superposition for PDADMA-PAA complexes and suggested that the complexation pH does not affect the relaxation mechanism.^90^

## Swelling Due to Salt and Solvent

Water clearly has an influence on the glass transition and dynamics of PEMs and PECs because both take up water, leading to swelling. Water uptake into PEMs and PECs has been studied extensively as a function of numerous factors such as salt type, ionic strength, temperature, humidity, and the terminating layer during and after assembly.^21,68,69,73,91−96^ Swelling is often caused by changes in polyelectrolyte chain conformation, breaking intrinsic ion pairs to form extrinsic ion pairs, and electrostatic repulsion. For example, osmotic pressure can influence the postassembly swelling of PEMs at low ionic strengths.^96^ In general, swelling is considered in terms of overall bulk swelling and swelling with respect to water microenvironments.

In this section, we discuss our contributions to understanding the swelling response of PEMs to different salts, solvent additives, and temperature using experimental techniques such as Fourier transform infrared (FTIR) spectroscopy,^21^ QCM-D,^57,68,91,92^ and MDSC^73,76^ but also MD simulations.^76^ To study the effects of salt specifically on PEM swelling, a number of variables have been explored: co-anion and co-cation type,^69,91^ monovalent and divalent co-ions,^68^ and salt effects during assembly and postassembly.^21,92^ We have also addressed the effect of swelling by the presence of mixed solvents.^97^

### Swelling of PEMs in Monovalent and Divalent Salt Solutions

The swelling of PEMs in different salt solutions is well documented, but in the mid-2010s, there was still a knowledge gap as to why certain PEMs swelled in relation to co-cation and/or co-anion type. To examine this, we explored the swelling responses of PDADMA/PSS PEMs assembled in 0.5 M NaCl that were immersed in KBr solutions after assembly.^91^ Upon exposure, the sodium and chloride co-ions that were compensating for extrinsic ion pairs would exchange to some degree with the contacting salt solution. For example, PEMs demonstrated the preferential uptake of K^+^ and Br^–^ ions and the release of Na^+^ and Cl^–^ ions upon immersion in KBr solutions.^91^ Comparing the swelling behavior of PEMs in KBr to that of NaCl, KCl, and NaBr, we observed that the co-anion type had a higher impact on ion exchange and swelling in the PDADMA/PSS PEM, with Br^–^ causing the most swelling.

Four swelling regimes were identified as the postassembly KBr salt concentration was increased from 0 to 2.0 M for the PDADMA/PSS PEMs.^91^ In the first regime, from 0–0.001 M KBr, excess swelling of up to 120% occurred as a result of electrostatic repulsion of the uncompensated PDADMA from neighboring charge sites. In the second regime, from 0.01 to 0.5 M KBr, the PEMs contracted slightly as the postassembly salt concentration began to match the assembly concentration and an adequate number of counterions are present for compensation. In the third regime, from 0.5 to 1.6 M KBr, swelling occurred proportionally to the postassembly concentration as a result of increased electrostatic screening and the conversion of intrinsic to extrinsic ion pairs. In the fourth regime, >2.0 M KBr, excessive charge screening led to deconstruction of the film. In our later works, we observed similar swelling regimes for PDADMA/PSS PEMs under different measurement methods.^21,92^

Divalent ions are known to cause bridging between the polyelectrolyte chains,^98,99^ which may cause very different swelling behaviors for PEMs as compared to those of monovalent ions. To better understand this, we studied the effects of three divalent salt cations and anions, CaCl_2_, MgCl_2_, and Na_2_SO_4_, on PDADMA/PSS PEMs using QCM-D.^68^ The results indicate that Mg^2+^ ions were superior doping agents because of their larger hydration shells as compared to those of Ca^2+^. These observations are characteristic of the Donnan exclusion/inclusion principle and the hydration properties of the ions interacting with the PEMs. On the other hand, the Na_2_SO_4_ concentration did not have a strong effect on swelling. This points to the dominance of the divalent salt cation over the divalent salt anion in the swelling and doping of PEMs because PDADMA is present in excess within the PEM.

### Swelling of PEMs in Mixed Solvents

Previous studies explored the swelling of PEMs in aqueous solutions of different ionic strengths. However, studies have shown that solvent quality could also impact PEM growth and swelling.^96,100,101^ To understand solvent effects, we explored the swelling/deswelling behavior of PEMs postassembly in ethanol– or urea–water mixtures.^97^ On a molecular level, MD simulations showed that urea and ethanol had significant but opposite effects on both PDADMA and PSS solvation and interactions. For an individual PSS chain, ethanol preferentially accumulated at the hydrophobic backbone with a higher water accumulation at the charged sulfonate groups. For an individual PDADMA chain, ethanol distributed in a manner associated with water layering. On the other hand, urea was not influenced by hydrophobicity and evenly accumulated around both PSS and PDADMA. When complexed, the introduction of both ethanol and urea pushed the polyelectrolytes apart, but ethanol caused a smaller change in backbone separation through counterion condensation as compared to urea which formed a persistent solvation shell. These simulation results were confirmed using zeta potential measurements for individual polyelectrolyte solutions and QCM-D for complexed films. Specifically, PSS/PDADMA PEMs exposed to urea solutions of increasing concentration experienced greater swelling that those exposed to ethanol solutions.

Besides solvent effects, we have explored the combined effect of salt and temperature on the hydration of PDADMA/PSS PEMs using attenuated total reflectance (ATR) FTIR spectroscopy.^21^ Water uptake in response to postassembly exposure to varying concentrations of NaCl in deuterated water HOD and varying temperatures was monitored through changes in the oxygen–deuterium (OD) stretch peak. Overall, increasing the temperature only slightly reduced the PEM’s water content, whereas the addition of salt led to more significant swelling, consistent with the previous observations.^68,91^ Taken together, PEMs swell in response to solvent, temperature, and ionic strength but also ion species. Additionally, the nature of water within the PEM, i.e., bound vs bulk-like, can strongly influence properties, as discussed below.

## Water Microenvironments in Polyelectrolyte Complexes and Multilayers

Water plays a crucial role in PECs as water can transform a PEC from the dry, brittle state to a rubbery state when hydrated. As a result, many studies have been carried out to understand the role of water in complexation and resulting properties. The literature presents varying ideas, definitions, and characterization classifications for the various states of water within a PEC, but overall, the important factor is to consider the molecular view of water microenvironments present in a PEC. In a review by Koehler et al., the authors summarized their works on water microenvironments in polyelectrolyte multilayers determined from neutron reflectometry.^93^ They identified two main types of water, namely, “void water”, which occupies pores within the PEM LbL film but does not contribute to thickness changes, and “swelling water”, which mainly contributes to thickness changes.^93,102,103^ Comparable results have been found using MD simulations showing “bulk” water molecules within water channels in PDADMA/PSS PECs and “surface” water molecules in direct contact with the PE chains.^104^ The findings assigned microstates as “void water” having bulk-like water characteristics and “surface water” having constrained dynamics indicating a bound, confined state. Hence, changes in the surface water influence the chain interactions.

**Figure 10:**
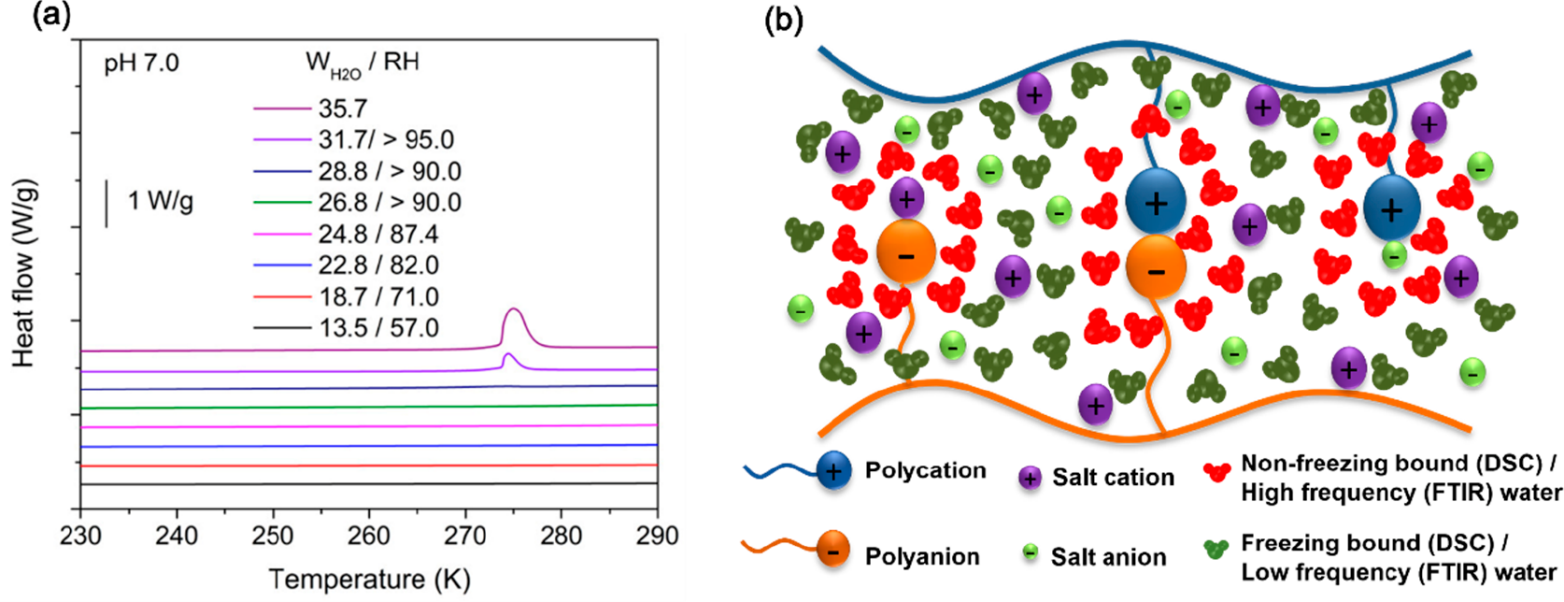
(a) DSC heat flow curves for PAH/PAA PECs at pH 7.0 with varying water content. Adapted with permission from ref (88). Copyright 2021 American Chemical Society. (b) Water microenvironments in polyelectrolyte complexes and multilayers as identified by DSC and ATR-FTIR spectroscopy. Reproduced from ref (21) with permission from the Royal Society of Chemistry.

In 2018, we investigated water microenvironments by monitoring the changes in the freezing temperature of water within PDADMA/PSS PECs as the PEC hydration increased from 18 to 30 wt % water by DSC measurements.^73^ Three possible water microenvironments were considered. At hydration levels of <30 wt % water, no freezing peak was identified in the DSC heat flow curves, indicating the existence of “non-freezing bound” water. Corresponding MD simulations indicated that for up to 30 wt % water, the vast majority of water molecules remained immobilized due to the strong interactions with the PE charge groups. However, at 30 wt % water, a small melting peak in the DSC data was identified from 255 to 270 K, lower than the melting temperature of pure water at 273 K. This indicated the existence of “freezing but bound” water within the PEC. “Freezing free” water with a melting peak at 273 K was not identified in the studied hydration range, indicating that all water had some binding interaction with the ion pairs. Similarly, freezing bound water was identified in complexes of weak polyelectrolytes PAH/PAA at hydration levels >28.8 wt % water as shown in Figure 10a.^88^

In a similar approach, the water microenvironments in complexes containing both weak and strong polyelectrolytes (PAH/PSS and PDADMA/PAA) were compared.^76^ For PAH/PSS PECs, at low hydrations of <30 wt % both nonfreezing bound and freezing bound water were identified, and no freezing free water was observed. At hydrations from 30–38 wt % water, freezing free water became evident and increased with the added water content while the nonfreezing bound water content decreased. On the other hand, all water in the PDADMA/PAA PEC existed in the nonfreezing bound state. MD simulations explained this difference in that PAA–water hydrogen bonding in PDADMA/PAA PECs was stronger than PSS–water hydrogen bonding in PAH–PSS PECs. In general, we showed that the distribution of water into the different microenvironments is closely related to the interplay between the PE–PE and PE–water interactions strengths.

We have probed water microenvironments in PDADMA/PSS PEMs also by ATR-FTIR spectroscopy for varying concentrations of NaCl-HOD solutions.^21^ By deconvoluting the OD stretching peak to subcontributions, three possible water microenvironments were identified. The “high frequency” water represents water that is tightly bound at polyelectrolyte ion pairs, the “low frequency” water represents water that is more loosely bound to polyelectrolyte ion pairs, and the “bulk” water corresponds to water that is free of polyelectrolyte ion pair interactions. At all studied temperatures and ionic strengths, no bulk water peak was identified, corroborating our prior findings from DSC.^73,76^

In these prior DSC^73,76,88^ and ATR-FTIR spectroscopy^21^ studies, we considered water microenvironments (Figure 10b) in relation to polyelectrolyte ion pairs, but the studies could not distinguish between water at intrinsic or extrinsic ion pairs. In our latest QCM-D study, we developed a method to create this distinction and estimate the number of water molecules specifically associated with intrinsic ion pairs. Here, PSS/PDADMA PEMs were assembled and then exposed to NaCl or KBr solutions of varying ionic strength. At postassembly salt concentrations >0.5 M, a linear dependence existed between the total amount of water per intrinsic ion pair and the postassembly salt concentration. The number of water molecules associated with intrinsic ion pairs, “*i”*, was obtained from the slope of the line, and the hydration coefficient (representing the hydrating power of the salt) was obtained from the intercept. We observed higher values of *i* in KBr-assembled PEMs because KBr is a more chaotropic and thus a more effective doping agent as compared to NaCl.

## Conclusions and Outlook

This Feature Article provided a perspective on our group’s contributions to the general understanding of the glass transition, dynamics, swelling, and water effects in PEMs and PECs. The past decade has brought about remarkable advances in revealing the connections among these factors, but a complete understanding is lacking. Therefore, we believe that the study of the relaxation of PEMs and PECs remains a rich area for future exploration.

For example, our prior work focused mostly upon simple polyelectrolyte system, with the neglect of hydrophobic interactions. However, hydrophobic interactions appear to have a nuanced influence on the polyelectrolyte complex. In one example, Kazi Sadman et al.^105^ demonstrated that increasing hydrophobicity of the polycation had a nonlinear influence on mechanical properties and increased resistance to salt resistance. On the contrary, Yang et al.^106^ reported that increasing hydrophobicity decreased the critical salt concentration. These contradictory results show us an unclear understanding and expectation of the influence of hydrophobic interaction on the thermal transition of PECs, making it an interesting area for future exploration. Besides hydrophobic interactions, hydrogen-bonding interactions should also be considered. Li et al.^107^ investigated the influence of nonelectrostatic interactions on the phase behavior with various pH values for PAA and PAH. Besides, the effect of hydrogen bonding on the PEC phase behavior was examined with increasing concentrations of urea to progressively inhibit hydrogen bonding. Similarly, we recently showed^97^ that polymer–solvent and polymer–polymer interactions could be altered by introducing urea or another solvent additive with a different dielectric constant, such as ethanol or others. Besides these factors, we propose that a closer examination of water microstates and intrinsic ion pairing should be carried out. For example, we proposed in the section above that water could shift contact intrinsic ion pairs to solvent-separated intrinsic ion pairs, but direct evidence does not yet exist.

Overall, the knowledge gained with respect to the dynamics of PEMs and PECs should have a long-lasting impact on their processing and use in future applications in energy, health, and the environment. With this new understanding, we envision that PEMs and PECs could be specifically tuned by the selection of assembly conditions and water content to yield desired mechanical properties and performance.

## Supporting Information Available

Linear fitting of ln(number of intrinsic ion pair/number of water molecules) vs −1/*T*_g_ for poly(allyamine hydrochloride)–poly(acrylic acid) complexes prepared at complexation pH values equal to 3.5, 5.5, 7.0, and 9.0 (PDF)

## Abbreviations

PEMs: polyelectrolyte multilayers
PECs: polyelectrolyte complexes
PEs: polyelectrolytes
*T*_g_: glass-transition temperature
PDADMA: poly(diallyldimethylammonium)
PSS: poly(sodium 4-styrenesulfonate)
PAH: poly(allylamine hydrochloride)
PAA: poly(acrylic acid)
QCM-D: quartz crystal microbalance with dissipation
MDSC: modulated differential scanning calorimetry
NAA: neutron activation analysis
LbL: layer by layer
*T*_tr_: thermal transition
^1^H NMR: nuclear magnetic resonance
MD: molecular dynamics
*y*^+^ or *y*^–^: cation or anion doping level
IP: ion pair
τ: relaxation time
DSM: dynamic structure model
RH: relative humidity
*σ*_DC_: DC conductivity
TTS: time–temperature superposition
*a*_*T*_: horizontal temperature shift factor
*E*′: storage modulus
TTWS: time–temperature–water superposition
MSD: mean square displacement
*ϕ*_W_: volume fraction of water
*ϕ*_f_: free volume fraction
*E*″: loss modulus
tan(δ): ratio of loss modulus to storage modulus
KWW: Kohlrausch–Williams–Watts
FTIR: Fourier transform infrared
ATR: attenuated total reflectance
HOD: deuterated water
OD: oxygen–deuterium
