# Supplementary material for: A Perspective on the Glass Transition and the Dynamics of Polyelectrolyte Multilayers and Complexes
Source: Langmuir. 2023 Oct 11;39(42):14823–39. doi: 10.1021/acs.langmuir.3c00974 (PMC10863056; doi:10.1021/acs.langmuir.3c00974)
Supplement: Supplementary file 1 — la3c00974_si_001.pdf [file la3c00974_si_001.pdf]

Supporting Information for

A Perspective on the Glass Transition and the Dynamics of Polyelectrolyte Multilayers and Complexes

*Hongwei Li,[1] Suvesh Manoj Lalwani,[1] Chikaodinaka I. Eneh,[1] Tamunoemi Braide,[1] Piotr Batys,[2] Maria Sammalkorpi,[3,4,5] Jodie L. Lutkenhaus[1,6]**

1. Artie McFerrin Department of Chemical Engineering, Texas A&M University, College Station, Texas 77843, United States

2. Jerzy Haber Institute of Catalysis and Surface Chemistry, Polish Academy of Sciences, Niezapominajek 8, 30-239 Krakow, Poland

3. Department of Chemistry and Materials Science, Aalto University, P.O. Box 16100, 00076 Aalto, Finland

4. Department of Bioproducts and Biosystems, Aalto University, P.O. Box 16100, 00076 Aalto, Finland

5. Academy of Finland Center of Excellence in Life-Inspired Hybrid Materials (LIBER), Aalto University, P.O. Box 16100, 00076 Aalto, Finland

6. Department of Materials Science and Engineering, Texas A&M University, College Station, Texas 77840, USA

Corresponding Author

*Email address: jodie.lutkenhaus@tamu.edu

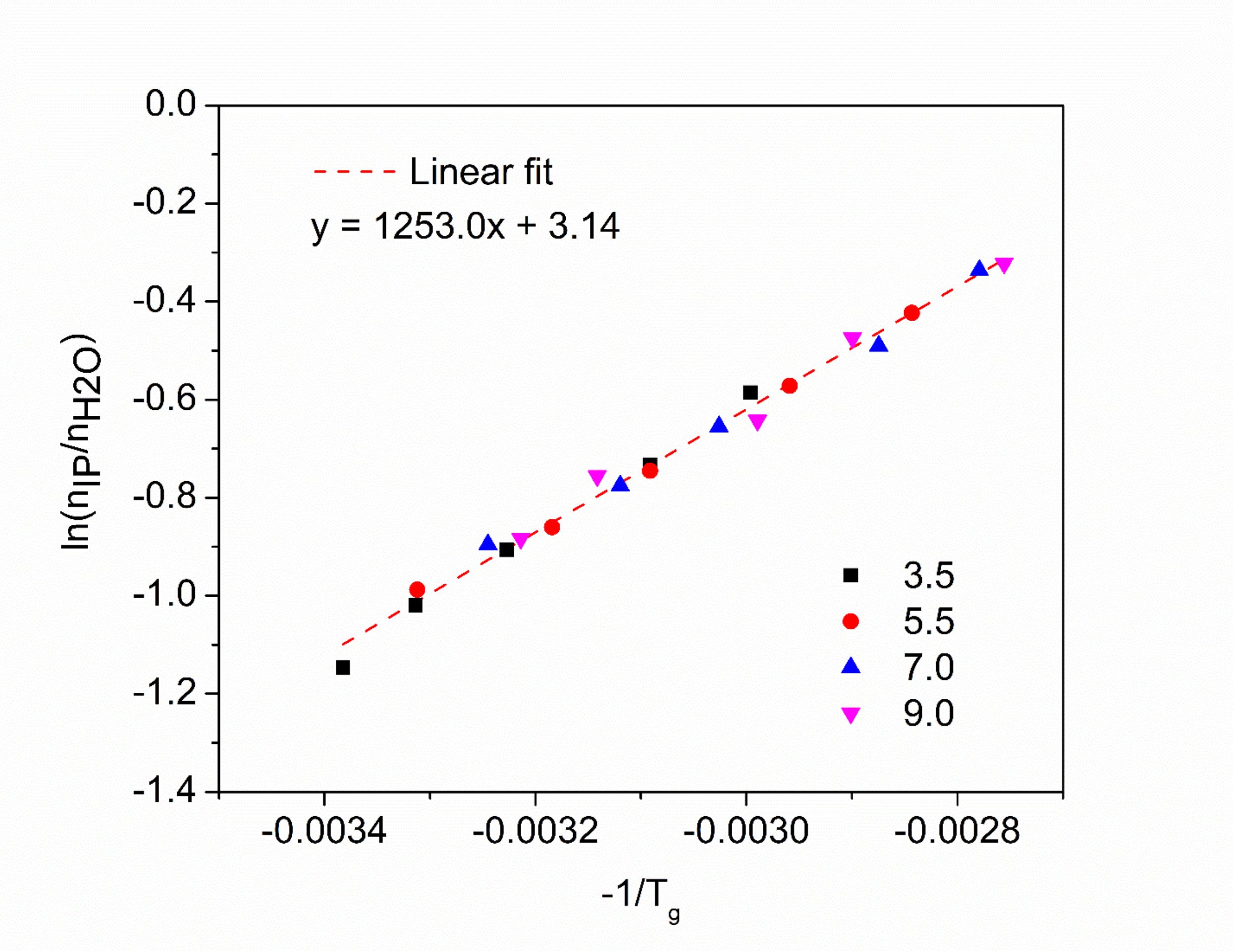

Figure S1 Linear fitting of ln(number of intrinsic ion pair/number of water molecules) vs -1/Tg for poly(allyamine hydrochloride) (PAH) – poly(acrylic acid) (PAA) complexes prepared at complexation pH values equal to 3.5, 5.5, 7.0 and, 9.0. The values for this figure were taken from reference 1.[1]

REFERENCES

1. Zhang, Y.; Li, F.; Valenzuela, L. D.; Sammalkorpi, M.; Lutkenhaus, J. L., Effect of Water on the Thermal Transition Observed in Poly(allylamine hydrochloride)–Poly(acrylic acid) Complexes. *Macromolecules* **2016,** *49* (19), 7563-7570.
